# Biological Activities of Tetrodotoxin-Producing *Enterococcus faecium* AD1 Isolated from Puffer Fishes

**DOI:** 10.1155/2015/973235

**Published:** 2015-08-24

**Authors:** Tu Hoang Khue Nguyen, Huu Ngoc Nguyen, Dat Van Nghe, Kim Hoang Nguyen

**Affiliations:** ^1^School of Biotechnology, Hochiminh International University, Vietnam National University, Quarter 6, Linh Trung Ward, Thu Duc District, Hochiminh City, Vietnam; ^2^Retina Center, 1715 N. George Mason Drive, Suite 101, Arlington, VA 2220, USA

## Abstract

Puffer fishes were collected from the central sea in Vietnam from spring to summer season. The eggs were incubated in MRS broth that was used to test the toxicity in mice and isolate the lactic acid bacteria community that could produce tetrodotoxin (TTX). Thin layer chromatography (TLC) and high performance lipid chromatography (HPLC) were used to detect and quantify TTX. As a result, *Enterococcus faecium* AD1 which was identified by biochemical test and 16S rRNA analysis could produce TTX 0.3 mg/mL when cultured in MRS broth. The bacterium was optimized for TTX production and gave 0.18 mg/mL, 0.07 mg/mL, and 0.15 mg/mL in media prepared from the meat-washing water of freshwater fishes (*Pangasius bocourti*, *Oreochromis* sp.) and sea fish (*Auxis thazard*), respectively, that are also hopeful to answer some poisoning cases related to eating fishes. *Enterococcus faecium* also showed the wide antimicrobial activities on yeast, Gram-negative and -positive bacteria. Extracted exopolysaccharide (EPS) that reacted with 2,2-diphenyl-1-picrylhydrazyl to give IC_50_ at 5 mg/mL equaled 11 mg/mL ascorbic acid which could show effects on Hela-6 and Hep G2 using sulforhodamine B test. *Enterococcus faecium* can be claimed as a promising source in tetrodotoxin and biological compounds.

## 1. Introduction

Tetrodotoxin (TTX) is the most well-known marine toxin, but TTX has been proven to be useful compound in pharmaceutical science. For example, TTX might be used as neuroprotective agent for ischemic damage of brain followed by strokes [1], renoprotective agent [2], and antinociceptive agent [3]. Up to now, TTX has been isolated from marine animals, including xanthid crab* Atergatis floridus* [4], the blue-ringed octopus* Octopus maculosus* [5], starfish* Astropecten polyacanthus* [6], and several species from various countries. In addition to animals, TTX-producing bacteria were reported to have been isolated from marine or freshwater sediment [7–9]. All the TTX-bearing animals were infected by TTX-producing bacteria lying within their bodies. Bacterium that produced TTX was reported in* Vibrio*,* Pseudomonas*,* Alteromonas*,* Bacillus*,* Aeromonas*,* Flavobacterium*,* Microbacterium arabinogalactanolyticum*,* Serratia marcescens*, and* Providentia rettgeri* [10, 11]. It has been known for many years that toxicity of the ovaries of puffer fish is the most potent one although the toxicity is also detected in other organs.

Because of TTX toxicity, looking for the TTX-producing source is always required so that the environmental safety is ensured. Most of researches found the TTX-producing microorganisms were not GRAS (generally recognized as safe), the study focused on lactic acid bacteria. Therefore, selection of ovary for isolation of TTX-producing lactic acid bacteria will be interesting. Lactic acid bacteria are important organisms recognized for their fermentative ability as well as their health and nutritional benefits [12]. They produce various compounds such as organic acids, diacetyl, hydrogen peroxide, and bacteriocin or bactericidal proteins during lactic fermentations [13–15]. The antimicrobial properties of lactobacilli are of special interest in developing strongly competitive starter cultures for food fermentation.

Lactobacilli exert strong antagonistic activity against many microorganisms, including food spoilage organisms and pathogens. Bacteriocins are antimicrobial proteinaceous compounds that are inhibitory towards sensitive strains and are produced by both Gram-positive and Gram-negative bacteria [16–20]. Bacteriocins have widespread potential applications in preservation for improving the safety and quality of foods. At present, there are many antibiotic-resistant pathogens; therefore, it is necessary to identify, develop, or redesign antibiotics to fight the multidrug resistance bacteria. Up to now, bacteriocins hold great promise as the next generation of antimicrobials. Beside facing drug resistance, the diseases which are due to the “oxidative stress” are the challenges that should be prevented.

Oxidant stress is initiated by free radicals, which produced aerobic metabolism in the body, and can cause oxidative damage of biological macromolecules such as proteins, lipids, and DNA in healthy human cells [21–23]. These changes contribute to oxidative stress that is among the major causative factors in the induction of many chronic and degenerative diseases including atherosclerosis, ischemic heart diseases and diabetes mellitus, cancer, immunosuppression, neurodegenerative disease, ageing [24–28], coronary heart disease, and Alzheimer's disease [29–32]. All human cells protect themselves against free radical damage by enzymes such as superoxide dismutase and catalase or compounds such as ascorbic acid, tocopherol, and glutathione [33]. However, these protective mechanisms occurred by various pathological processes, and antioxidant supplements are necessary to combat oxidative stress. Therefore, the interest in the natural compounds with strong antioxidant and anticancer properties has steadily been increasing.

From the above stated reasons, the TTX-producing lactic acid bacteria were isolated in puffer fish and then optimized the ability of TTX production of bacteria in media prepared from freshwater fishes (*Pangasius bocourti*,* Oreochromis* sp.) and sea fish (*Auxis thazard*). Then, this strain was used to study antimicrobial, radical scavenging, and anticancer activities to find out the potential lactic acid bacteria for pharmaceutical science.

## 2. Materials and Methods

### 2.1. Sample Preparation and Bacteria Cultures

Puffer fishes were collected at the sea in the center of Vietnam. No specific permit was required for the described field studies. The female fish weight was around 600 g, kept in ice, and transported to the lab. The female fishes were washed with 70% and dissected to isolate the ovaries from abdomen by sterilized knife and scissor. The ovaries (3 g) were homogenized carefully and incubated directly in modified MRS broth. Samples were incubated for 48 hours in 5% CO_2_ at room temperature. Then, the cultures were checked for microorganims on MRS agar.

### 2.2. Isolation and Identification of* Enterococcus faecium*

One mL of culture was aseptically spreaded on MRS agar plates and was incubated at 25°C for 3–5 days. Eventually, the forming colonies were transferred onto another new MRS agar plates until discrete colonies were obtained. Each discrete colony was further subcultured for several times to ensure that a pure culture was obtained. The pure cultures were also characterized using bioassay for toxicity test. The toxin was extracted and purified for thin layer chromatography and high performance liquid chromatography. Then, the toxic strain was identified by biochemical characterization based on the ability of utilization of different carbon sources by API 50CHL (bioMerieux, Lyon, France). Then, molecular characterization using 16S rRNA sequencing analysis was performed. The forward primer was faec1 (5′-ttggagagtttgatcctggctcaggac-3′) and the reverse primer was faec2 (5′-gctgctggcacgtagttagccgtggct-3′). The PCR reaction was performed as follows: 95°C for 5 min; 30 cycles of 95°C for 20 s, 50°C for 20 s, and 72°C for 3 min; and a final extension at 72°C for 10 min. The PCR product was stored at 4°C. The PCR product was purified and sequenced. The homology comparison of the almost 16S rRNA gene sequence was performed using the NCBI BLAST database.

### 2.3. Extraction and Purification of Tetrodotoxin from Bacterial Cultures

The isolation and purification procedures of TTX from bacterial cultures were basically the same as those used for puffer fish tissues. Each broth culture was centrifuged at 4,000 rpm for 15 min to remove the bacterial debris. The supernatant was mixed with an equal volume of water-washed activated charcoal under agitation and filtered through a Buchner funnel. The charcoal on the funnel was thoroughly washed with distilled water and TTX was eluted for 3 times with 3 volumes of 1% acetic acid in 20% aqueous ethanol [10, 17, 34, 35]. The eluate was heated at 100°C for 10 min and then cooled and centrifuged to separate debris. The eluate was evaporated, freeze-dried, and then dissolved in 10 mL of 0.03 M acetic acid for gel filtration. Eluted TTX was used for further toxicity bioassay, thin layer chromatography, and high performance liquid chromatography.

### 2.4. High Performance Liquid Chromatography (HPLC) for Tetrodotoxin Detection

The extracted samples were applied to HPLC in order to detect and quantify the TTX existence by UV detector. The Shimadzu HPLC system with RP 18 (250 × 4.6 mm; 5 *μ*m) column was used as stationary phase. The mobile phase was prepared by mixing of 0.01 M sodium heptanesulfonic and methanol at ratio 90 : 10. The flow rate was 1 mL/min at 30°C; wavelength was 205 nm. The authentic TTX (Tocris, UK) at concentration 0.2 mg/mL was applied.

### 2.5. Bioassay for Toxicity Detection

Mice were injected intraperitoneally with extraction [36]. The toxicity was determined by the death time of each injected mouse. The death time was recorded at the grasping breath of mouse. It was recommended not to use weight more than 23 g and reuse mice.

### 2.6. Thin Layer Chromatography (TLC) for Tetrodotoxin Detection

TLC analysis method was carried out [34] with a little modification. The eluted samples were applied on TLC silica gel 60 F256—Merck's product. The mobile system was* n*-butanol-acetic acid-distilled water (5 : 1 : 2) prepared in TLC chamber [10]. The spots were observed under UV light (256 nm) and compared to TTX standard.

### 2.7. Optimization of TTX-Producing Condition in Bacteria

As the concerning on the effects of media to optimize the TTX production abilities of bacteria, the study had used the media prepared from fresh fishes (*Pangasius bocourti*,* Oreochromis*) and sea fish (*Auxis thazard*). Each test was performed in triplicate. After preparing all media, they were autoclaved at 121°C in 15 minutes. The starting inoculum would be recorded in colony forming unit (CFU) per milliliter and the finishing inoculum as well [37]. The TTX production was detected by bioassay, TLC, and HPLC.

### 2.8. Antimicrobial Activity Test

The indicator pathogens such as* Candida albicans* ATCC 10231,* Salmonella typhi* ATCC 19430,* Pseudomonas aeruginosa* ATCC 27853,* Staphylococcus sciuri* ATCC 29061,* Micrococcus luteus* ATCC 10240, and* Staphylococcus aureus* ATCC 25923 were used in the study. All pathogens (10^6^ cfu/mL) were cultured in 200 mL of LB for 18 h.

### 2.9. Preparation of Cell-Free Supernatant (CFS) of Isolated Strain for Antimicrobial Test

The isolated strain (10^6^ cfu/mL) was cultured in 500 mL of MRS broth for 24 h. Cell-free supernatant (CFS) of isolated strain was prepared following the method constructed by Ogunbanwo et al. [38]. The culture was centrifuged at 10,000 rpm for 20 minutes at 4°C. Then, the CFSs were collected and stored in 4°C for further use.

### 2.10. Test of Antimicrobial Activities of Isolated Strain

The strain was tested for their antimicrobial activities to the indicator pathogens by well-agar diffusion assay of Tagg JR [39]. Indicator pathogens were spread onto LB agar plates. The wells with 5 mm diameter were carved on the plates. Then, they were filled up with 100 *μ*L of CFS. The plates were incubated at 37°C for 24 h and examined the antimicrobial activities by observing the zones of inhibition appearing around each well. The experiment was triplicated.

### 2.11. Effect of pH on the Growth of Isolated Strain

In order to study the effect of incubation temperature on growth and antimicrobial agent production, the amount of 10^5^–10^6^ cfu/mL was inoculated into buffered MRS broth and was adjusted at different pH values (1, 1.5, 2, 2.5, 3, 4, 5, 6.5, 7.5, 8, and 8.5) before cultivation [39]. The bacterial growth was checked by counting the survival on MRS agar. Then, the growth percentage of bacteria in different pH was based on the number of survival bacteria and the number of starting bacteria. Each assay was performed in triplicate.

### 2.12. Bacteriocin Precipitation

The strain was grown in MRS broth at 37°C in 48 h. Supernatant was collected after centrifugation at 10,000 rpm for 15 min at 4°C. Ammonium sulfate was added slowly to the cell-free culture supernatant with constant stirring to achieve the concentration of ammonium sulfate (40%). The mixture was centrifuged at 10,000 rpm for 30 min at 4°C. The collected pellet was dissolved in phosphate buffered saline (PBS), pH 7.4, and was ready for the antimicrobial test. The remaining supernatant fraction was subsequently adjusted with ammonium sulfate to 60 and 80%. The precipitant in each ammonium sulfate fraction was dissolved in PBS (pH 7.4) and then was ready for antimicrobial test. Bacteriocin was checked on sodium dodecylsulphate-polyacrylamide gel electrophoresis (SDS-PAGE; 15% discontinuous gel). The molecular weight of the fractionated proteins was compared with standard markers (Bio-Rad).

### 2.13. Preparation of Polysaccharides

The isolate was cultured in (MRS) and incubated at 37°C under aerobic conditions. Cultures were centrifuged at 10,000 rpm for 30 min to remove debris. The culture supernatant was added with three times volume of absolute cold ethanol (EtOH) and left overnight. The precipitant was collected after centrifugation at 10,000 rpm for 30 min. The obtained pellet was resuspended in distilled water and further precipitated by adding three times volume of cold ethanol. The overnight solution was centrifuged to collect the water-soluble exopolysaccharides (EPS). The crude EPS was dried at 60°C to a constant weight. EPS stocks were dissolved in distilled water and then filtered through the 0.22 *μ*m pore-size filters (Millipore, Bedford, MA) before use.

### 2.14. Antioxidant Activity Using DPPH Radical Scavenging Assay

In order to perform the 2,2-diphenylpicrylhydrazyl (DPPH assay), amounts of 4.3 mg of DPPH were dissolved in 3.3 mL methanol in a test tube [40]. Solution was protected from light by covering the test tubes with aluminum foil. 150 *μ*L of the above solution was added to 3 mL methanol. This solution was measured at 517 nm on UV spectrophotometer. Methanol was used as blank. For standard (ascorbic acid) curve, the aliquots of different concentration range were prepared. After 50 *μ*L of tested EPS (10 mg/mL) and standard ascorbic acid in the various concentration were diluted with methanol up to 3 mL, 150 *μ*L DPPH was added. All these samples were taken after 12 h and measured at 517 nm on UV-visible spectrometer (Shimadzu, UV-1601, Japan). The DPPH free radical scavenging activity was calculated using the following formula:

(1) $$\begin{array}{c} \frac{\left(1-A_{s}\right)}{A_{c}}\times 100, \end{array}$$

where *A*_*s*_ and *A*_*c*_ are the absorbance of control and sample, respectively.

### 2.15. Anticancer Activity Test

In order to perform antitumor activity test, the crude EPS sample was prepared according to the above prescribed procedure. The sulforhodamine B (SRB) assay was used in the study according to the method of Longo-Sorbello with a slight modification [41]. Each cancer cell line was seeded in a 96-well plate (1.0 × 10^4^ cells per well). After 24 h of incubation, the test samples (115.3 × 10^6^ cfu/mL of cell-free supernatant and 20 mg/mL of crude EPS) were added to the cancer cells, 5% CO_2_ for 48 h at 37°C. For this incubation time, no significant differences were observed in the pH of medium. Thereafter, 50 *μ*L of 50% TCA (4°C) was added to each well containing 200 *μ*L of medium to reach a final concentration of 10% TCA in each well and let the 96-well plate for 1 h at 4°C to allow cell fixation. After 1 h of incubation, the culture medium in each well was removed and the plate was gently washed with water (200 *μ*L/well) five times and dried at room temperature for 12–24 h. 0.2% SRB (w/v) solution was added after the time for incubation to each well and left at room temperature for 5–20 min. Then, washing the plate with 1% acetic acid was performed five times in order to remove unbound SRB. Drying the plate for 12–24 h before adding 200 *μ*L Trizma-base 10 mM in order to solubilize bound SRB is necessary. The last step is plating the 96-well plate on a plate shaker for at least 10 min. Absorbance was measured at 492 nm and 620 nm using an enzyme-linked immunosorbent assay plate reader (Molecular Devices, Sunnyvale, CA, USA). DMSO was used as a negative control. The percentage of viable cells was calculated based on the difference of the optical density of experimental group and control group with the optical density of control group. The SPSS 16.0 software (SPSS Inc., Chicago, IL, USA) was used to calculate the means and standard deviations in any experiments involving triplicate analyses of any samples. The statistical significance of any observed difference was evaluated by one-way analysis of variance (one-way ANOVA), using the Bonferroni multiple comparisons test.

## 3. Results and Discussion

### 3.1. Identification of* Enterococcus faecium*

**Figure 1:**
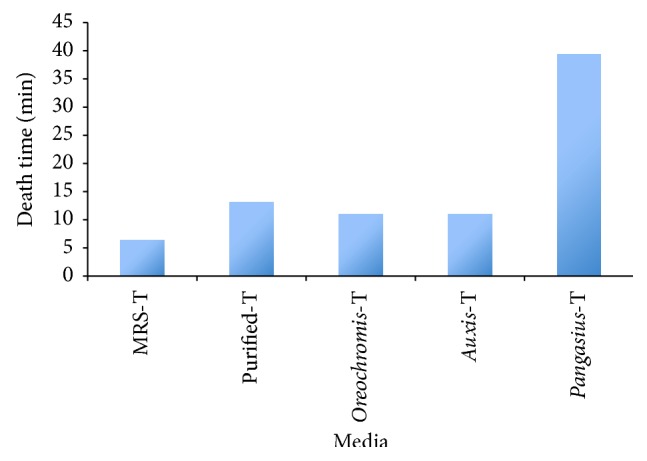
Death time of mice injected with different bacterial cultures prepared from MRS, purified tetrodotoxin from MRS culture,* Oreochromis* sp.,* Auxis thazard*, and* Pangasius bocourti*.

After detecting the toxicity of the puffer fish ovaries in MRS, the isolation of TTX-producing bacteria was done. There were 5 dominant bacterial strains isolated from ovaries of the puffer fish. Only one of them was determined to be toxic in mouse bioassay. The bacterium could produce 1.5–3 *μ*g ranging within 30–80 MU (mouse per unit) in 100 mL of broth medium. The death time occurred in a short time at average time of 6 mins (Figure 1). The culture showed that the mouse toxicity was purified and each purified fraction was used to test toxicity (Figure 1).

**Figure 2:**
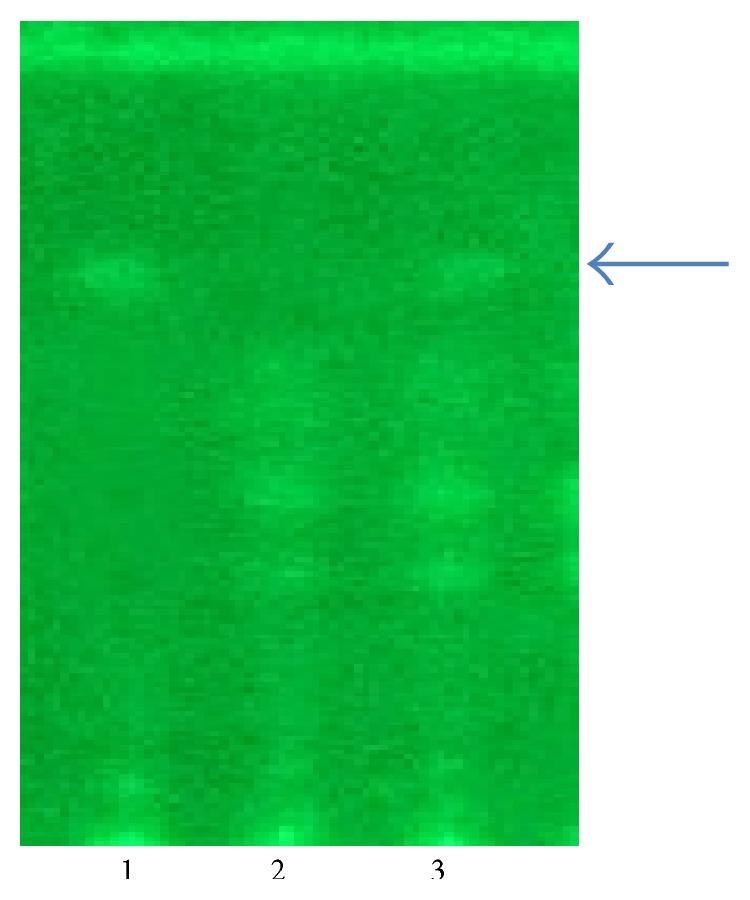
Thin layer chromatography analysis on tetrodotoxin detection. 1: standard tetrodotoxin, 2: MRS medium, and 3: extract from bacterium. The arrow showed tetrodotoxin spot.

In the toxicity detection using mouse bioassay, the relationship between dose and time to death is used to quantify each sample. This relationship is approximated by log⁡MU = 2.3log⁡(1 + *T* ^−1^), where MU is the number of mouse units of tetrodotoxin injected and *T* is time to death in hours. The mouse assay has been traditionally used, but it is unsuitable as a market test. There are other disadvantages such as the variation in mouse weight that must be limited involving a large breeding colony of the mice, and the death time relationship to dose is nonlinear. Therefore, the study combined TLC and HPLC analyses for qualification and quantification. The TTX production of the bacterium was detected on TLC (Figure 2).

**Figure 3:**
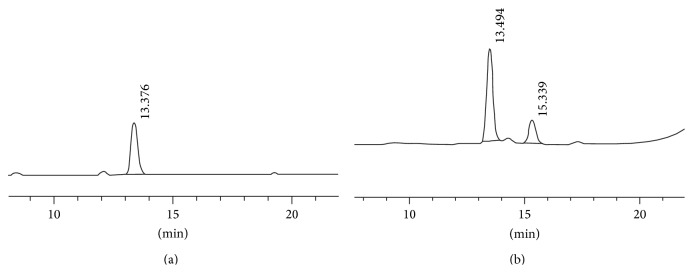
High performance liquid chromatography analysis of tetrodotoxin. (a) Standard tetrodotoxin. (b) Purified tetrodotoxin from MRS culture.

The spots of the standard TTX and purified toxin of the isolated strain had the same *R*_*f*_ value (0.65) under UV illumination. In comparison with the negative control as the media, the bacterium could produce the spots with the same *R*_*f*_ of the TTX standard; this bacterium could produce suspected TTX. By HPLC analysis, the sample prepared from bacteria in this study also showed the peak at retention time (13.494 min) that was similar to the retention time of the peak of standard TTX (13.376 min) (Figure 3). Based on mouse bioassay test and TLC and HPLC analysis, this bacterium could produce TTX.

After determining the bacteria that could produce toxin by bioassay, TLC, and HPLC, the bacterium was identified by biochemical tests and 16S rRNA sequence analysis. The bacterium was found to be a facultative anaerobe which can utilize and produce acid by fermenting d-glucose, mannitol, rhamnose, d-sorbitol, mannose, d-galactose, d-fructose, and 10% lactose. The partial 16S rRNA sequence was 540 bp deposited in DDBJ (Accession number AB828395). The partial 16S rRNA gene sequence was analyzed by using NCBI BLAST, showing 99% of the homology to* Enterococcus faecium* Aus004 and 97% of the homology to* Enterococcus faecium* DSM 20477. There was no information of tetrodotoxin-producing* Enterococcus faecium* announced before. Therefore, the isolated strain was identified* Enterococcus faecium* AD1.

This study is the first report of TTX-producing* Enterococcus faecium* isolated from puffer fishes in Vietnam and the world. The discovery of TTX-producing* Enterococcus faecium* supports the theory that TTX is originated from the microorganisms found inside the TTX-bearing organisms. Normally,* Enterococcus faecium* itself could not produce TTX except this bacterium surviving in TTX in puffer fishes. This indicated that there was an interaction of puffer fish and bacteria. Interestingly, the bacterium which could produce TTX in puffer fishes could produce TTX in some kinds of fish like TTX-producing* Providentia rettgeri* [10]. TTX is conventionally obtained by the extraction and purification from toxic organs of the puffer fishes that gave an extremely low TTX yield, about 1 ton of toxic puffer ovaries required to yield 1 g of pure TTX [35]. Study on the biosynthesis of TTX directly from TTX-producing* Enterococcus faecium* AD1 in vitro is much more economical and efficient than the traditional method. Besides, when* Enterococcus faecium* AD1 produces TTX, TTX formulation as probiotic will be hopeful application in using TTX without further TTX purification that affects the living environment. Thus in order to obtain mass production of TTX from TTX-producing bacteria in vitro, it is necessary to develop the optimum medium composition, such as the temperature, pH, salinity, rate of shaking, and light intensity, as well as the effective methods of isolating TTX from the culture media (batch or continuous methods). In this study, we focused on the medium composition originated in freshwater fishes and sea fishes.

### 3.2. Optimization of TTX Production in* Enterococcus faecium* AD1

All the extracts from* Pangasius bocourti*,* Oreochromis *sp., and* Auxis thazard* were used for mouse bioassay (Figure 1). As seen in Figure 1, TTX produced in medium prepared from* Pangasius bocourti* was the lowest. The TLC analysis for the TTX production in the fish media was also performed. The spots of the standard TTX and those of the extracts had the same *R*_*f*_ value (0.65) under UV illumination in TLC analysis. By HPLC analysis, the sample prepared from* Pangasius bocourti*,* Oreochromis *sp., and* Auxis thazard* in this study also showed the peak at retention time of 13.330, 12.802, and 13.324 min, respectively. The retention time in different samples was evaluated using standard deviations and maximum response. Using the United States Pharmacopoeia (USP) method, the tailing factors should not exceed 2 and the peak response is acceptable. The standard deviation was less than 2%. The other separate peaks are not of concern to this study. Those retention time values were close to the retention time of the peak of standard TTX (13.376 min) which meant that these fish cultures could be the good substrates for* Enterococcus faecium* AD1 to produce TTX. The yield produced by this bacterium in fish media was summarized in Table 1.

From Table 1, the trends of TTX production have partly shown the suitable condition in natural life. The medium prepared from* Oreochromis *sp. (0.18 mg/mL calculated on the final product in 0.03 M acetic acid) was higher than in* Auxis thazard* (0.15 mg/mL). The hypothesis is pointing that this TTX-producing bacterium can take place in any kinds of fishes fed in lake or in sea. For media prepared from freshwater fishes, with the inoculum of 10^14^ cells in 10 mL media, TTX amount of cultured sample in* Oreochromis *sp. equaled 0.18 mg/mL, which was larger than in* Pangasius bocourti* (0.07 mg/mL). It makes sense when making the relation to many poisoned cases in consuming* Oreochromis *sp.; in this situation,* Oreochromis *sp. tissues are the ideal places for bacteria to take place, especially for* Enterococcus* as we analyzed above. TTX production in different fish media was interesting that might be the significant difference in the contents of calcium, sodium, and potassium in these fishes. These ions might be affected on the TTX-producing pathway of bacterium. More studies should be performed to clarify the mechanism. In practice, these kinds of fishes did not contain TTX. It was thought that there were immune responses to TTX in these alive fishes. The study will perform many experiments to study the poisoning ability in puffer fishes and other kinds of fishes infected with TTX-producing bacterium to bring up the detoxifying mechanisms. For TTX-producing strategies, to apply on many fields such as medical sciences and pharmaceuticals, we have to produce on large scale, so the economic point of view is more important. Meat-washing medium prepared from freshwater is the waste of fish industry; we can make use of it for targeted purposes.

### 3.3. Antibiotic Activities

**Figure 4:**
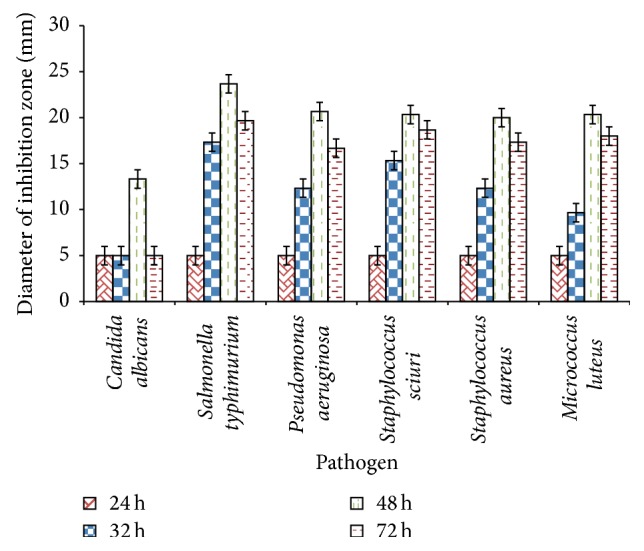
Antimicrobial activities of* E. faecium* in pathogens in different incubation times.

The antibiotic activities of* E. faecium* AD1 were shown in Figure 4.* E. faecium* AD1 could inhibit* Candida albicans*,* Salmonella typhi*,* Pseudomonas aeruginosa*,* Staphylococcus sciuri*,* Staphylococcus aureus*, and* Micrococcus luteus*. As seen in Figure 4, the activity showed strong effect at 48 h culture. When bacterium was cultured less than 48 h, the exponential phase was reached and there were small amounts of antimicrobial agents produced. Otherwise, for the 72 h culture, the antimicrobial activity became lower than the 48 h culture which meant that there were some degraded antimicrobial agents; for example, the proteins could inhibit the pathogens, but they were lysed by protease produced by* E. faecium* AD1 itself.

The activities on pathogens in different stages of culture were not similar that might be the antagonist activities of different antimicrobial agents probably happening in the culture stages. More study should be done in the future to clarify these actions. By bacteriocin precipitation, two bacteriocins that had the estimated molecular weight of 4 kDa and 4.5 kDa could be collected and showed antimicrobial activities.

### 3.4. Effect of Medium pH on Bacteria Growth

**Figure 5:**
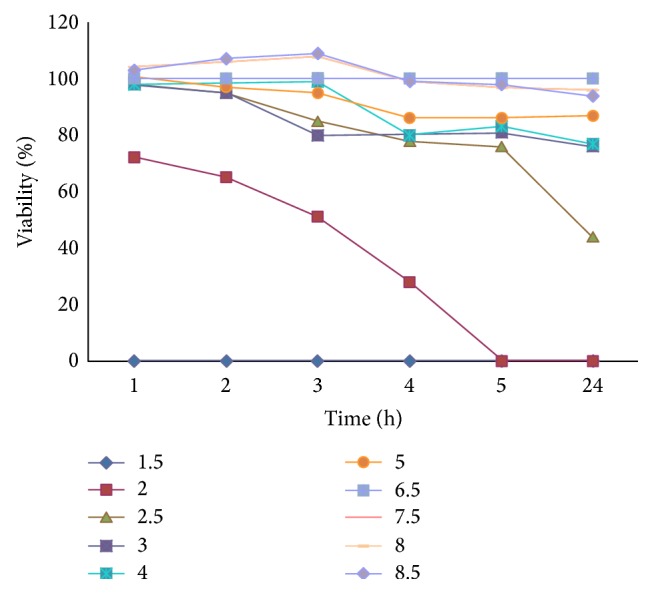
Effects of pH on growth of* Enterococcus faecium*.

Besides the culture period that was studied in antimicrobial activity, medium pH was also important factor that affects cell growth as well as bacteriocin production. The bacteriocin was treated with a wide range of pH values from 1.5 to 8.5. This bacterium could survive at pH 2 in 5 h. However, it could survive at pH from 2.5 to 8.5 (Figure 5). With the advantage of the adaptability to a wide pH range, this bacterium could be used in different formulations. Moreover, this bacterium could survive in gastrointestinal tract that was of benefit to orally pharmaceutical formulations for tetrodotoxin formulation from* Enterococcus faecium* without further purification to prevent the TTX contamination to environment.

### 3.5. DPPH Radical Scavenging Assay

In this present study, the antioxidant activity of the crude EPS was done by using the DPPH scavenging assay. The IC_50_ of crude EPS (5 mg/mL) equaled 11 mg/mL ascorbic acid to give the antioxidant activities according to the standard curve (*y* = 1.7204*x* + 43.475, *R*2 = 0.9993). From the results of antioxidant activity, it was recognized clearly that the bacterium could produce the highest amount of antioxidant activity.

### 3.6. Anticancer Activities

As illustrated in Table 2, CFS and EPS were effective to Hela and Hep G2 cancer cells. Depending on the different mechanisms like receptor binding ability to cancer cell lines, the cytotoxicity percentage of each sample was also different. For Hela cancer cell, the cytotoxicity percentage of EPS was 90.42 ± 2.965. In case of Hep G2 cancer cell, the cytotoxicity percentage of crude EPS was 70.28 ± 3.125. These results indicated that the EPS isolated from* E. faecium* constituted the major fraction that inhibits the proliferation of cancer cells. The CFS gave the lower activities in Hela (40.15 ± 5.469) and Hep G2 (60.51 ± 6.568) as compared to EPS because EPS in the study was concentrated from 100 mL of cell-free supernatant that was directly used to test cancer cells. More studies should be done so far. Remarkably,* Enterococcus faecium* AD1 isolated could be the potent source for bioactivity studies.

## 4. Conclusions

The study has identified a TTX-producing* Enterococcus faecium* AD1 and optimized the TTX production in cheap source and also suggested that the fishes could be infected with TTX. However, because of the immune response, they will or will not prevent TTX in their life. The study will uncover the mechanism to prevent TTX infection so far. Moreover, TTX-producing* Enterococcus faecium* AD1 could survive in different pH and produced antimicrobial, antioxidant, and anticancer activities that might supply a potential probiotic used to yield TTX in reducing pain in cancer without further TTX purification as well as producing the activities for cancer and pathogen treatment.

## Figures and Tables

**Table 1:** TTX concentration (mg/mL) produced by *E. faecium* in media prepared from different fishes.

Samples	Concentration (mg/mL)
Standard	0.2
*Auxis thazard*	0.15
*Pangasius bocourti*	0.07
*Oreochromis *sp.	0.18

**Table 2:** Inhibitory effects of cell-free supernatant and exopolysaccharide on the growth of two human cancer cell lines.

Cancer cell line	% cytotoxicity	
	Cell free supernatant	Crude EPS
Hela	40.15 ± 5.469	90.42 ± 2.965
Hep G2	60.51 ± 6.568	70.28 ± 3.125
